# Spatial Distribution of Polycyclic Aromatic Hydrocarbon Contaminants after Hurricane Harvey in a Houston Neighborhood

**DOI:** 10.5696/2156-9614-11.29.210308

**Published:** 2021-03-02

**Authors:** Garett T. Sansom, Katie R. Kirsch, Gaston A. Casillas, Krisa Camargo, Terry L. Wade, Anthony H. Knap, Erin S. Baker, Jennifer A. Horney

**Affiliations:** 1 Department of Environmental and Occupational Health, Texas A&M School of Public Health, College Station, Texas, USA.; 2 Department of Epidemiology and Biostatistics, Texas A&M School of Public Health, College Station, Texas, USA; 3 Interdisciplinary Faculty of Toxicology, Texas A&M University, College Station, Texas, USA; 4 Geochemical and Environmental Research Group, Texas A&M University, College Station, Texas, USA; 5 Department of Chemistry, North Carolina State University, Raleigh, North Carolina, USA; 6 Epidemiology Program, University of Delaware, Newark, Delaware, USA

**Keywords:** polycyclic aromatic hydrocarbon, soil, environmental justice

## Abstract

**Background.:**

Hurricane Harvey made landfall along the Texas Gulf Coast as a Category 4 hurricane on August 25, 2017, producing unprecedented precipitation that devastated coastal areas. Catastrophic flooding in the City of Houston inundated industrial and residential properties resulting in the displacement and transfer of soil, sediment, and debris and heightening existing environmental justice (EJ) concerns.

**Objectives.:**

The primary aim of this study was to evaluate the presence, distribution, and potential human health implications of polycyclic aromatic hydrocarbons (PAHs) in a residential neighborhood of Houston, Texas following a major hurricane.

**Methods.:**

Concentrations of PAHs in 40 soil samples collected from a residential neighborhood in Houston, Texas were measured. Spatial interpolation was applied to determine the distribution of PAHs. Potential human health risks were evaluated by calculating toxicity equivalency quotients (TEQs) and incremental excess lifetime cancer risk (IELCR).

**Results.:**

Total priority PAH concentrations varied across samples (range: 9.7 × 10^1^ ng/g-1.6 × 10^4^ ng/g; mean: 3.0 × 10^3^ ng/g ± 3.6 × 10^3^ standard deviation). Spatial analysis indicated a variable distribution of PAH constituents and concentrations. The IELCR analysis indicated that nine of the 40 samples were above minimum standards.

**Conclusions.:**

Findings from this study highlight the need for fine scale soil testing in residential areas as well as the importance of site-specific risk assessment.

**Competing Interests.:**

The authors declare no competing financial interests.

## Introduction

Environmental justice (EJ) communities are disproportionately impacted by environmental pollution and inadequately protected from these impacts by policies and regulations.^1^ Excess exposure to environmental pollutants due to the proximity to toxic waste sites, polluting industries, and municipal waste facilities occurs disproportionately in EJ communities.^2^ This is due to the historical practice of locating polluting facilities in low-income and minority communities, which are less able to object because of their lack of political leverage or access to system-level power structures.^3^ Residents of EJ communities bear an undue burden of detrimental health outcomes as a result of these exposures, such as respiratory disease,^4,5^ cardiovascular disease,^6^ adverse pregnancy outcomes,^7,8^ cancers^9,10^ and other chronic illnesses.^11,12^ While numerous studies have focused on chronic exposures and their associations with health outcomes, there is a growing need to understand how acute environmental exposures associated with natural or technological disasters may affect already polluted EJ neighborhoods through the environmental mobilization of contaminants.^13^

The Harrisburg Manchester Super Neighborhood is located adjacent to the Houston Ship Channel, a 50-mile-long waterway linking the City of Houston to the Gulf of Mexico.^14^ The Neighborhood is also adjacent to one of the world's largest petrochemical refineries, a major highway, and a rail yard. The Houston Ship Channel, often referred to as the petrochemical corridor, is known to contain pesticides from agricultural run-off, indicator bacteria from sewage, and other toxic chemicals.^15^ Residents have frequently expressed concerns that storm surge and flooding associated with hurricanes, tropical storms, and inland precipitation could transport contaminants from the HSC and nearby petrochemical refining and processing facilities, landfills, and transportation infrastructure to their neighborhoods.^16–18^ In addition to approximately 80 direct deaths and $180 billion in damages,^19^ when Hurricane Harvey became the wettest tropical cyclone to ever impact the US^20^ and between 30 and 60 inches of rain resulted in extensive flooding across the region, the potential health effects associated with the mobilization of contaminants was a major concern to residents. With the frequency of events with more than 20 inches of precipitation increasing by 1% from 1981 to 2000, and forecasted to increase 18% between 2081 and 2100,^21^ the risks that these events will impact residents of Texas is increasing. Residents of Houston Ship Channel communities are highly vulnerable to both environmental and public health impacts that could result from pollutant redistribution following extreme flooding events.

Polycyclic aromatic hydrocarbons (PAHs) are known pollutants that have been associated with EJ communities in general and within Houston Ship Channel neighborhoods in particular.^22,23^ Polycyclic aromatic hydrocarbons are formed through the incomplete combustion of organic compounds and can result from the burning of biomass in cooking,^24,25^ forest fires,^26^ or from anthropogenic sources including petrochemical and coal manufacturing.^27^ Although PAHs are ubiquitous in the environment, they have also been linked with numerous adverse human health effects.^28^ In the 1970s, the United States Environmental Protection Agency (USEPA) classified 16 PAHs as priority pollutants due to their known toxicity to humans and occurrence in the environment.^29^ Over the last 50 years, these 16 PAHs have served as proxies for total PAH contamination, although some limitations have been noted,^30^ in part due to the PAH exposure literature's focus on occupational exposures.^31,32^ More recently, a growing body of evidence has linked non-occupational exposures to PAHs to potential health effects, such as exposure through recreational activities, food, and after disasters.^23,33–35^ Our study expands upon these by assessing increased risk of exposure to PAHs through fate and transport during flooding. Since PAHs can bind to particulate matter,^36^ they can be redistributed in soils within floodplains, changing exposure opportunities.^37^ Therefore, rapid disaster response research is needed to improve our understanding of potential risks and protect the health of the public after these types of disasters.^38^ Post-disaster data may also provide baseline values for future assessments of health impacts during normal weather conditions and after the frequent natural and technological disasters that impact the Houston region and its many industrial facilities.^39^

**Abbreviations**
*EJ*: Environmental Justice
*IELCR*: Incremental Excess Lifetime Cancer Risk
*TEF*: Toxicity Equivalence Factor
*TEQ*: Toxicity equivalency quotients

## Methods

**Figure 1:**
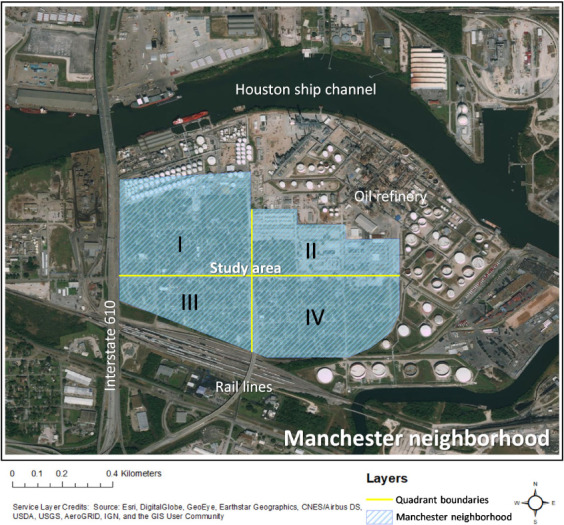
Four quadrants of the Manchester neighborhood of Houston used for evaluation in this study

The geographically compact neighborhood of Manchester, part of the Harrisburg Manchester Super Neighborhood, is located adjacent to the Houston Ship Channel, Interstate 610, and a 24-line railyard *(Figure 1).* The historic community comprising the Harrisburg Manchester Super Neighborhood was established as a railroad trading post in the early 1860s,^40^ preceding congressional approval for a port of delivery at Houston, Texas on July 14, 1870.^41^ The neighborhood is known to have an unequal burden of exposure to pollution^42–44^ and associated health risks.^45^ Manchester is both physically and socially vulnerable to the impacts of disasters; 88% of the Super Neighborhood's residents are Hispanic/Latino with a median income one-third less than the City of Houston overall, and only 8% of residents have obtained a Bachelor's degree.^14^

### Sample Collection

In partnership with staff from Texas Environmental Justice Advocacy Services (t.e.j.a.s.) and residents of Manchester, teams of faculty, community engagement staff, and graduate students from the Texas A&M University Institute for Sustainable Communities (IfSC) and the Texas A&M University Superfund Research Center (SRC) collected sediment samples on September 1, 2017, one week after Hurricane Harvey made landfall. Team members donned powder-free nitrile gloves and used a clean metal trowel to collect samples from the top 2 to 3-cm of water-saturated soil, depositing the sample in a prepared 8 oz glass jar. The longitude and latitude of each sample location were recorded and all samples were placed in a cooler for transportation to the Geochemical and Environmental Research Group (GERG) at Texas A&M University. Upon arrival, samples were stored in −80°C freezers and then freeze dried in preparation for PAH extraction and quantification.

#### PAH Extraction

Sample extraction and analysis were performed in accordance with the standard protocol of GERG, as previously described.^22,42^ Extraction was performed with a Dionex ASE 200 Accelerated Solvent Extractor (ASE) operated at elevated temperature (100°C) and pressure (1500 psi) with a solvent mixture of dichloromethane/methanol (95/5%). After rinsing each ASE cell with dichloromethane/methanol, each cell was prepared by sequential insertion of a combusted filter, hydrochloric acid (38%)-activated granular copper (20–30 mesh), 8 g of freeze-dried sediment, and 100 ml of a quality control sample consisting of organics in marine sediment from the standard reference material (SRM-1941b).^46^ The PAH extracts were transferred into individual 250 mL volumetric flasks and granular copper and boiling chips were added. Flasks were placed in a water bath (60ºC) to facilitate solvent exchange to hexane and extract concentration via evaporation to a final volume of 1–2 mL. To ensure sample purity and minimize potential interference during analysis, concentrated extracts were purified using partially deactivated silica/alumina column chromatography.

#### Gas chromatography-mass spectrometry analysis

Quantitative analysis of the PAHs was achieved using a HP5890 gas chromatography system (HP5890, Hewlett Packard Company, Wilmington, DE) with MS detection (Agilent 5972, Agilent Technologies, Santa Clara, CA) in selected ion mode (SIM).^47^ Sample extracts were injected into a 0.60 m × 0.25 mm i.d. (0.25 μm film thickness) HP-5MS capillary column (Agilent HP-5MS, Agilent Technologies, Santa Clara, CA) with the initial injection port maintained at 285°C to achieve vaporization in advance of capillary column entry. The oven temperature was programmed to increase at a rate of 7°C/min from its initial temperature of 60°C to 310°C, which was maintained for a final holding time of 22 min. The USEPA 16 priority PAHs were quantified at a practical limit of 10 ng/mg extract.^48^

#### Data Analysis

The mean concentration and standard deviation of the 16 priority PAHs across the 40 soil samples were calculated. The total concentrations of PAHs in each soil sample were analyzed by summing the 16 priority PAH concentrations. To visualize the PAH soil concentrations in different locations in the Manchester neighborhood, the geographical region was split into four quadrants as shown in Figure 1. Quadrants I, II, and IV were located closer to the refinery, while I and III were next to Interstate 610, and III and IV were closer to the rail yard. Sampling was performed at 21 sites in Quadrant I, 3 in Quadrant II, 4 in Quadrant III and 12 in Quadrant IV. MetaboAnalyst (Montreal, Canada) was used to assess the total PAH concentration at each site and in the four different quadrants. The binary logarithm function (log_2_) was applied to log-transform individual PAH concentrations prior to analysis. Spatial interpolation was performed to further characterize the accumulation of the PAHs across the study area. Specifically, the concentrations of total PAHs, benzo(a)pyrene (BaP), pyrene, and naphthalene in each sample were mapped using ArcGIS.

Site-specific ecotoxicological risk was assessed using the toxicity equivalency quotient (TEQ) method, which provides a weighted estimate of the concentration of each PAH relative to the toxicity of BaP.^49,50^ Toxicity-weighted PAH levels were derived by multiplying the concentration of each individual PAH by its corresponding toxicity equivalence factor (TEF) *(Table 1).*^49,50^ The total BaP-TEQ was calculated by summing the toxicity-weighted values of the 16 priority PAHs.

**Table 1:** Average Concentrations of Priority Polycyclic Aromatic Hydrocarbons

Chemical	TEF	Concentration (ng/g)	Standard Deviation
Acenaphthene	0.001	2.0 × 10^1^	3.5 × 10^1^
Acenaphthylene	0.001	8.0 × 10^1^	2.4 × 10^2^
Anthracene	0.01	1.2 × 10^2^	2.9 × 10^2^
Benzo(a)anthracene	0.1	2.0 × 10^2^	2.3 × 10^2^
Benzo(a)pyrene	1	2.1 × 10^2^	2.3 × 10^2^
Benzo(b)fluoranthene	0.1	3.4 × 10^2^	3.9 × 10^2^
Benzo(g,h,i)perylene	0.01	1.5 × 10^2^	1.8 × 10^2^
Benzo(k)fluoranthene	0.1	1.1 × 10^2^	1.3 × 10^2^
Chrysene	0.01	2.7 × 10^2^	2.8 × 10^2^
Dibenzo(a,h)anthracene	1	3.1 × 10^1^	4.5 × 10^1^
Fluoranthene	0.001	5.4 × 10^2^	7.2 × 10^2^
Fluorene	0.001	2.2 × 10^1^	3.6 × 10^1^
Indeno( 1,2,3-c,d)pyrene	0.1	1.6 × 10^2^	1.8 × 10^2^
Naphthalene	0.001	3.7 × 10^1^	8.4 × 10^1^
Phenanthrene	0.001	2.2 × 10^2^	3.5 × 10^2^
Pyrene	0.001	4.7 × 10^2^	5.9 × 10^2^

A modified incremental excess lifetime cancer risk (IELCR) approach was next employed to evaluate the potential risk associated with the observed concentrations of PAHs in soils collected from Manchester. The IELCR for dermal exposures to soil has been previously utilized to assess potential cancer risk.^50^ The equation used to calculate IELCR is described by Yang *et al.*^50^ and shown below *(Equation 1):*

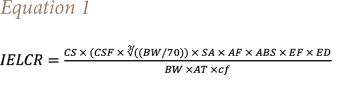
where CS is the total BaP-TEQ for each soil sample. The carcinogenic slope factor (CSF) used for dermal exposure to BaP was 25 (mg kg^−1^ day^−1^)^−1^. Dermal surface exposure (SA) was defined as 5000 cm^2^ day^−1^, the dermal adherence factor (AF) was 0.00001 kg cm^−2^, and the dermal absorption fraction (ABS) was 0.1 (unitless). To account for potential variability in PAH concentrations in soil subsequent to a major flooding event, the standard exposure frequency (EF) used to determine IELCR for dermal exposure was reduced from 350 days per year to 30 days per year, and exposure duration (ED) and average lifespan (AT) were drawn from standard values at 30 years and 70 years, respectively.^50^ For the same reason, body weight (BW) was defined as 70 kg and a conversion factor (cf) of 10^6^ was employed. A box and whisker plot was produced to represent the IELCR values (Microsoft Excel, Redmond, WA).

## Results

The total mean concentration of the priority 16 PAHs one week after Hurricane Harvey was evaluated for the 40 sites sampled *(Table 1).* The cumulative concentration of the 16 priority PAHs in each soil sample was variable with a range of 9.7 × 10^1^ ng/g to 1.6 × 10^4^ ng/g and a mean of 3.0 × 10^3^ ng/g ± 3.6 × 10^3^ standard deviation.

**Figure 2:**
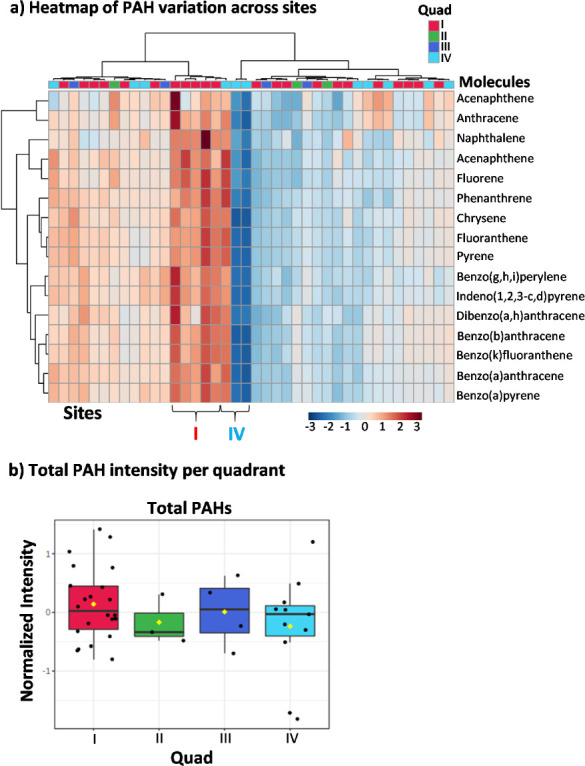
Polycyclic aromatic hydrocarbon site and quadrant concentrations. a) Heatmap assessment of PAH concentration for each site illustrated 5 high concentration sites in Quad I and one in Quad IV, while both low concentration areas were in Quad IV. b) The total concentration and c) specific PAH molecule concentration per quadrant showed Quad I to have the highest concentrations, while Quad II was found to be have the lowest PAH concentration.

Differences in PAH concentrations were observed across the 40 sites, with the five highest PAH concentrations present in Quadrant I and the two lowest in Quadrant IV, as shown by the heatmap in Figure 2a. The site with the highest concentration of the 16 priority PAHs was found at the northwest section of Quadrant I, nearest to Interstate 610 and the Houston Ship Channel, while the two lowest PAH areas were on the southeast section of Quadrant IV, farthest from this area. To assess exposure by quadrant, the total PAH concentration for the sites in each quadrant were averaged. As shown in the box plots in Figure 2b, Quadrant I exhibited the highest average level, while Quadrant II had the lowest average level. The individual concentrations of BaP, naphthalene and pyrene were further visualized in Figure 2c. In all cases, Quadrant II had the lowest average concentration of the three PAHs. However, the highest average concentrations of BaP were noted in Quadrant III, while Quadrant I was the highest for naphthalene and pyrene.

**Figure 3:**
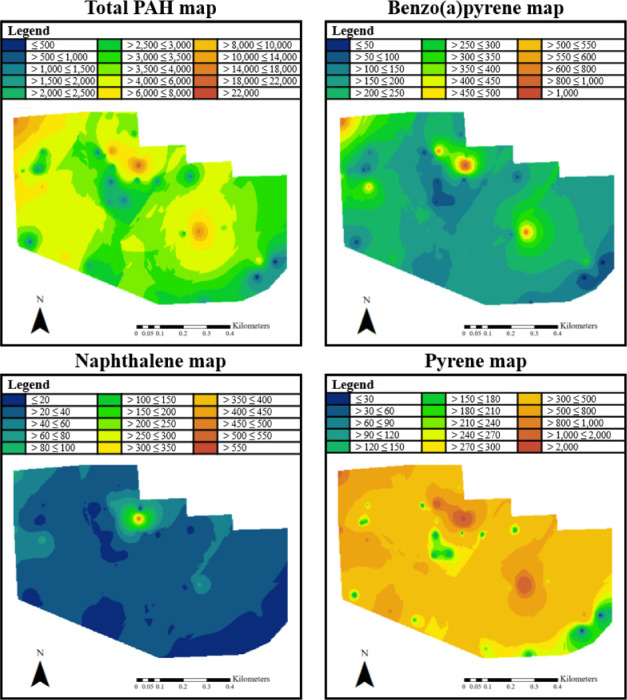
Spatial distribution of PAHs illustrates hot spots. Clockwise, the maps correspond to: total PAH concentration (top left), benzo(a)pyrene (top right), pyrene (bottom right) and naphthalene (bottom left), with all concentrations in ng/g.

To further characterize accumulation of PAHs in the study area, spatial interpolation was performed by mapping the site values onto the quadrants to identify hot spots *(Figure 3).* The results for Quadrant II were concordant with the average data in Figure 2, as it had lower total PAH concentration and individual PAHs levels. Quadrants I, III, and IV all had several hotspots noted for the 16 priority PAHs. Further assessment of the individual PAH spatial locations showed pyrene was the least variable through the Manchester region, while BaP was more localized to specific sites.

**Figure 4:**
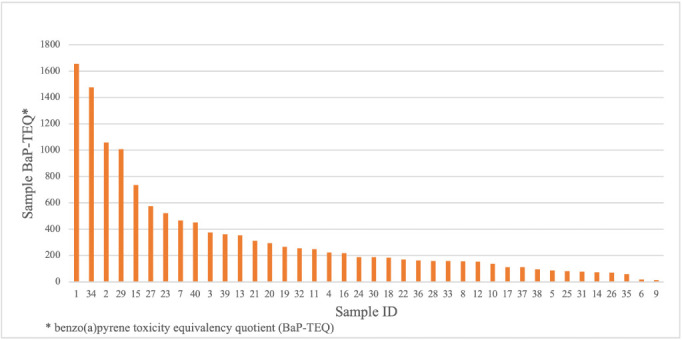
B(a)P-TEQ for the 16 priority PAHs within sample zones, Manchester, Houston, TX IELCR

The BaP-TEQ was calculated for each sample *(Figure 4).* Site-specific BaP-TEQ values varied between samples, ranging from 14.1 BaP-TEQ to 1,655.2 BaP-TEQ. The respective mean and standard deviation for all samples were 332.9 BaP-TEQ and 368.1 BaP-TEQ.

### IELCR

**Figure 5:**
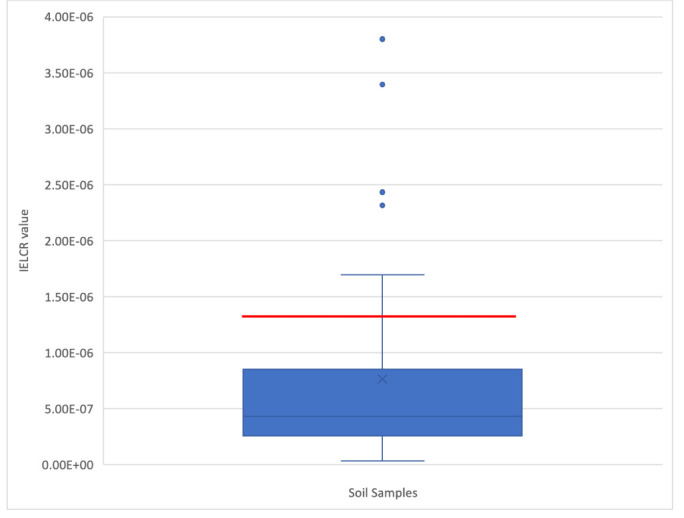
Box and whisker plot of IELCR values corresponding with soil samples collected from the Houston, Texas neighborhood of Manchester (N=40). The area delineated in red indicates an IELCR value of higher risk, as defined by the USEPA.^51^ IELCR

Guidelines provided by the USEPA^51^ have established that an IELCR between 10^−6^ and 10^−4^ indicates a higher potential risk of developing cancer. As illustrated in Figure 5, IELCR values ranged from 3.2 × 10^−8^ to 3.8 × 10^−6^ with a mean of 7.6 × 10^−7^. Nine of the 40 soil samples were found to have IELCR values in excess of the USEPA's lower bound for elevated cancer risk of 1.0 × 10^−6^.

## Discussion

According to soil contamination classification proposed by Maliszewska-Kordybach,^52^ nearly half of the Manchester neighborhood is experiencing at least weakly contaminated sections with some areas encountering heavy contamination. Spatial analytics revealed that PAH concentrations had a variable distribution throughout the site and were not isolated along the Houston Ship Channel, Interstate 610, or the 24-line railyard. While PAH distribution was seen throughout the neighborhood, there is one local hotspot across all PAH maps in the northern center, which is closest to the Houston Ship Channel. Total concentrations drop the further from these regions we sampled, with the southeast sections having the lowest concentration. With the heavy rainfalls experienced as part of Hurricane Harvey, the distributions may have been due to a combination of drainage management and the location of impervious surfaces throughout the neighborhood. The present study expands upon previous assessments showing that residents in flood prone regions may be at an increased risk of exposure to PAHs through fate and transport mechanisms.

This study has several important limitations. Street level shape files are not available for the City of Houston,^53^ requiring the organization of the Manchester neighborhood into quadrants to better understand concentrations and potential impacts of PAHs. While samples were rapidly acquired due to an ongoing partnership with community partners that informed site selection and sample collection, the timing of sample analysis means that post-disaster data related to potential acute pollution are not likely to be rapidly actionable to protect public health. Future studies should focus on improving understanding of baseline PAH concentrations to document local sources that can become focal points for PAH fate and transport in EJ neighborhoods during catastrophic flooding.

## Conclusions

The findings in this study demonstrate the need for finer scale testing to assess how PAHs are dispersed after hurricanes and floods. With 9 of the 40 samples containing concentrations above the minimum standard for increased cancer risks, this study provides evidence of the need for site specific risk assessment in EJ communities who are inequitably exposed to both environmental pollutants and natural disasters. More baseline data and best practices are needed to move forward more interdisciplinary, community-engaged research in EJ and other vulnerable communities that will experience more major flooding events in the decades to come.
